# NDRG2 Is a Novel p53-Associated Regulator of Apoptosis in C6-Originated Astrocytes Exposed to Oxygen-Glucose Deprivation

**DOI:** 10.1371/journal.pone.0057130

**Published:** 2013-02-22

**Authors:** Yan Li, Ning Xu, Lei Cai, Zijun Gao, Lan Shen, Qiaomei Zhang, Wugang Hou, Haixing Zhong, Qiang Wang, Lize Xiong

**Affiliations:** 1 Department of Anesthesiology, Xijing Hospital, the Fourth Military Medical University, Xi’an, Shaanxi, People’s Republic of China; 2 Department of Anesthesiology, Shaanxi Provincial Maternal and Child Health Hospital, Xi’an, Shaanxi, People’s Republic of China; 3 State Key Laboratory of Cancer Biology, Department of Gastrointestinal Surgery, Xijing Hospital of Digestive Diseases, The Fourth Military Medical University, Xi’an, Shaanxi, People’s Republic of China; 4 Institute of Molecular Biology and the State Key Laboratory of Cancer Biology, The Fourth Military Medical University, Xi’an, Shaanxi, People’s Republic of China; Duke University, United States of America

## Abstract

N-myc downstream-regulated gene 2 (NDRG2) has been documented to be a pro-differentiative and anti-proliferative gene in cancer research. Our previous study found a significant NDRG2 up-regulation in reactive astrocytes of penumbra after transient focal cerebral ischemia, which was parallel to the enhancement of TUNEL-positive signals. However, it is still uncertain whether NDRG2 participates in cellular apoptosis induced by ischemia-reperfusion injury in brain. In this study, we investigated the role of NDRG2 in cellular apoptosis induced by oxygen-glucose deprivation (OGD) in IL-6-differentiated C6 glioma cells. The results showed that NDRG2 was up-regulated and translocated from the cytoplasm to the nucleus after OGD exposure. NDRG2 over-expression exhibited an anti-proliferative effect and increased the Bax/Bcl-2 ratio after OGD exposure, while NDRG2 silencing promoted the cellular proliferation and attenuated the up-regulation of Bax/Bcl-2 ratio. The pro-apoptotic effect of p53 was verified by the results in which p53 silencing greatly reduced the percentage of OGD-induced apoptotic cells. p53 silencing also reduced the OGD-induced NDRG2 up-regulation. However, over-expression of p53 did not further improve the NDRG2 up-regulation. In conclusion, NDRG2 is a p53-associated regulator of apoptosis in C6-originated astrocytes after OGD exposure. These findings bring insight to the roles of NDRG2 in ischemic-hypoxic injury and provide potential targets for future clinical therapies on stroke.

## Introduction

N-myc downstream-regulated gene 2 (NDRG2), together with NDRG1, NDRG3 and NDRG4, belongs to the NDRG gene family, which is involved in cell proliferation and differentiation [1], [2]. Human NDRG2 was first identified from a normal human brain cDNA library by subtractive hybridization in 2001 (GenBank accession no. AF159092) [3]. NDRG2 was documented to be a pro-differentiative and anti-proliferative gene in previous cancer research. In adult tissues, the NDRG2 expression has been detected in salivary glands, brain, skeletal muscles, heart, liver as well as kidneys [4]. Because of its high expression in brain, NDRG2 was related to some important functions and pathophysiological process in central nervous system. In patients diagnosed of Alzheimer’s disease (AD), Mitchelmore *et al* observed that NDRG2 expression was up-regulated at both RNA and protein levels in cortical pyramidal neurons, senile plaques and cellular processes of dystrophic neurons [5]. Takahashi *et al* reported the down-regulation of NDRG2 expression in rat frontal cortex after long-term antidepressant and repeated electroconvulsive treatment [6]. In a previous study [7], we found a significant increase of NDRG2 expression in reactive astrocytes of penumbra after transient focal cerebral ischemia. And some NDRG2 signals also co-localized with TUNEL-positive cells. Based on these findings, we postulated that NDRG2 up-regulation in astrocytes might participate in cell apoptosis after cerebral ischemic-reperfusion (I/R) injury. However, the precise mechanisms still need to be elucidated.

Another tumor suppressor in cancer research, p53, is a transcription factor that stops the cell cycle and induces pro-apoptotic effect through modulating multiple target genes [8]. In murine brains, Wang *et al* reported that p53 deficiency played a central role in driving gliomagenesis [9]. Moreover, it also contributes to neuronal death after transient cerebral ischemic injury [10], [11], while delayed treatment with a p53 inhibitor could facilitate the endogenous neurogenesis and therefore improve the functional recovery [12]. Taken together the fact of pro-apoptotic effect of NDRG2 in tumor cells, we hypothesize that NDRG2 is involved in the p53-induced apoptosis in cerebral I/R injury.

The aims of this study were to determine (1) whether NDRG2 participates in cellular apoptosis induced by oxygen-glucose deprivation (OGD) in C6-originated astrocytes, and (2) whether NDRG2 is involved in the p53-induced apoptosis of astrocytes after OGD exposure. We found that NDRG2 contributed to OGD-induced apoptosis in C6-originated astrocytes and the OGD-induced up-regulation of NDRG2 was closely associated with p53. In addition, we also observed significant nuclear translocation of NDRG2 after OGD.

## Results

### Expression of NDRG2 was Up-regulated in C6-originated Astrocytes Exposed to OGD

**Figure 1:**
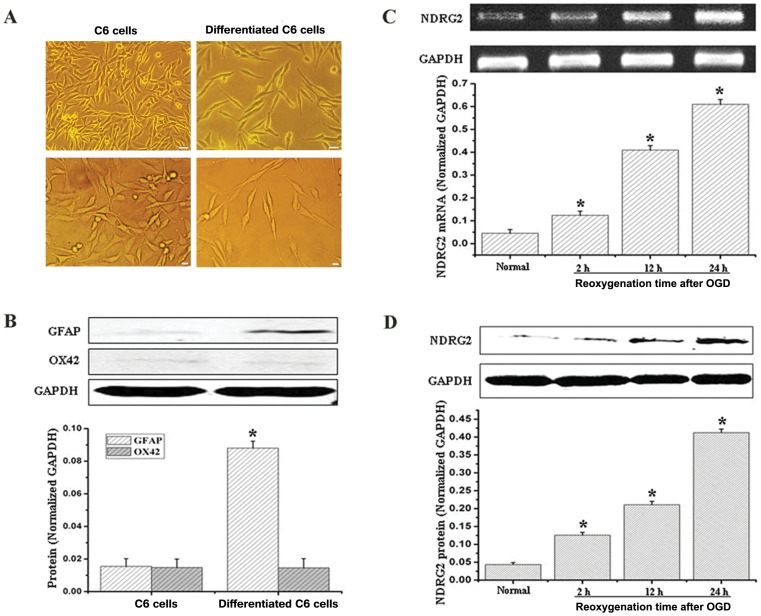
NDRG2 expression in C6 glioma cells after OGD. (**A**) The C6 glioma cells were subjected to RPMI 1640 medium in the absence or presence of 100 ng/ml IL-6 for 24 hours to induce an astrocyte-like differentiation. The upper (Scale bar = 20 µm) and lower row (Scale bar = 10 µm) showed different magnifications. (**B**) The IL-6-differentiated cells were verified by GFAP and OX42 in Western-blotting analysis. GFAP expression sharply increased in the differentiated cells, while OX42 expression maintained hardly detected. So it was astrocytes that we employed in the following experiments. (**C, D**) Both NDRG2 mRNA (C) and protein (D) were up-regulated after OGD exposure in a time-dependent way. NDRG2 mRNA and protein began to increase at 2 h after OGD, then reached a peak at 24 h. All data were presented as the mean ± SD of three independent experiments. Student’s *t* test, *P<0.05 *vs*. normal.

**Figure 2:**
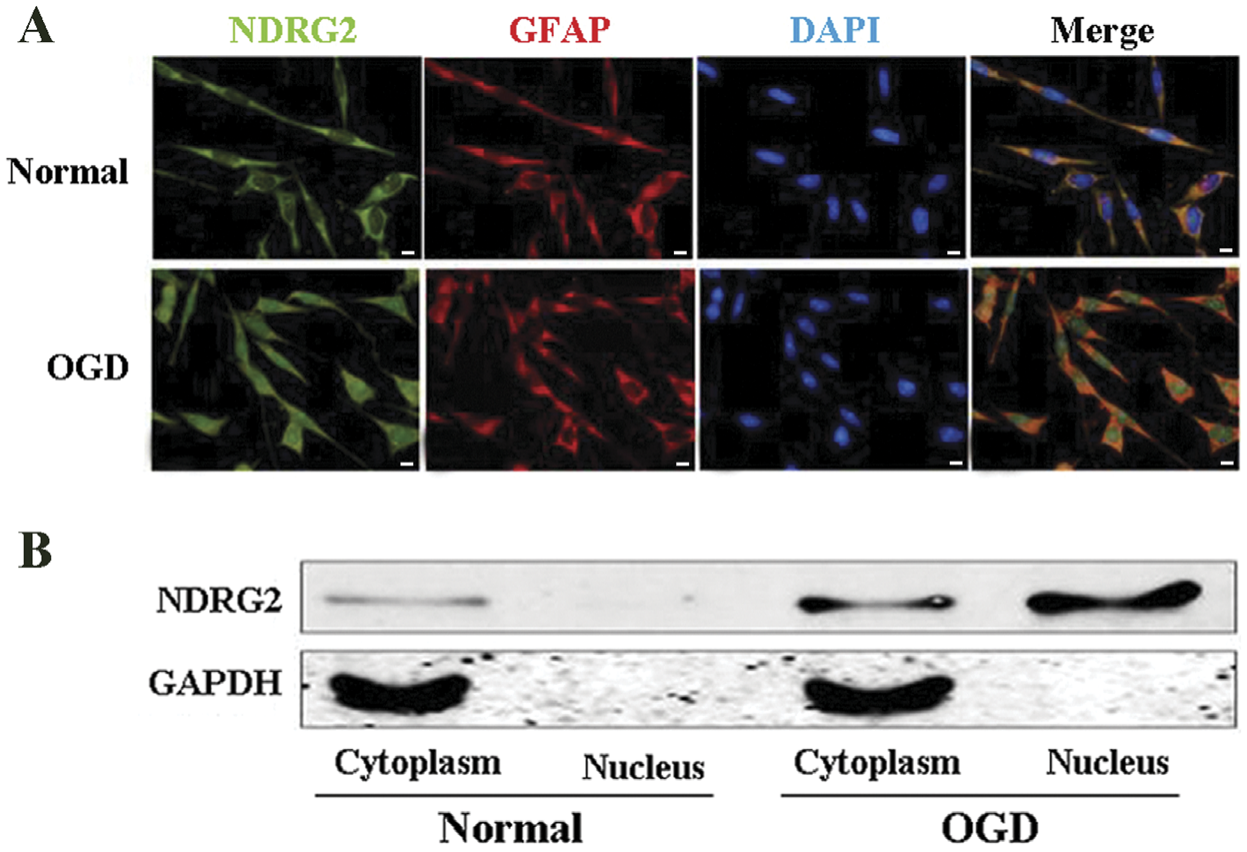
NDRG2 nuclear translocation after OGD exposure. (**A**) Immunofluorescent double-labeling staining of NDRG2 and GFAP showed the localization of NDRG2. NDRG2 is indicated in green, GFAP to mark the astroglial cytoplasm is indicated in red, and DAPI to mark the nucleus is indicated in blue. In normal astrocytes (upper row), NDRG2 expression overlapped with GFAP, but not with DAPI. In OGD-treated cells (lower row), NDRG2 expression overlapped with GFAP and DAPI simultaneously. Scale bar = 10 µm. (**B**) The NDRG2 expression in nucleus and cytoplasm extraction was measured with Western-blotting analysis. In normal astrocytes, NDRG2 could hardly be detected in nucleus. At the time of 24 h after OGD exposure, the NDRG2 expression both in the nucleus and in the cytoplasm sharply increased.

To investigate the role of NDRG2 in astrocytes, we employed the IL-6-differentiated C6 glioma cells as normal astrocytes. As shown in Fig. 1A, the morphology of C6 cells turned into an astrocyte-like pattern after incubation for 24 hours in IL-6 containing medium. Western-blotting analysis showed that the GFAP, a marker of astrocytes, was dramatically up-regulated in IL-6-differentiated C6 cells compared with naïve cells. At the same time, the expression of OX42, the microglia marker, could neither be detected in naïve nor in IL-6-treated cells (Fig. 1B). GFAP immunoreactivity also confirmed that C6 glioma cells were differentiated into astrocytes successfully by IL-6 treatment (Fig. 2A).

To test the changes of NDRG2 in astrocytes after OGD exposure, Western-blotting and RT-PCR were performed. The results showed that the expression of NDRG2 was significantly up-regulated in a time-dependent manner after OGD. Both NDRG2 mRNA (Fig. 1C) and protein (Fig. 1D) began to increase in 2 h after OGD and reached a peak in 24 h after OGD.

Taken together the results from this *in vitro* experiment and previous *in vivo* study, the time point of 24 h after OGD was chosen in the following experiments.

### NDRG2 was Translocated from the Cytoplasm to the Nucleus in Astrocytes after OGD

As shown in Fig. 2A, NDRG2 immunoreactivity did not overlap with DAPI, but with GFAP before OGD treatment, which suggested that the expression of NDRG2 was confined to the cytoplasm, rather than the nucleus in untreated astrocytes (Normal). However, it was observed that the signal of NDRG2 was markedly enhanced in nucleus at the time of 24 h after OGD exposure. The shift of NDRG2 expression indicated that NDRG2 was translocated from the cytoplasm to the nucleus, which was probably induced by the stress of OGD.

To further support the assumption of nuclear translocation of NDRG2 upon OGD exposure, a cell fraction assay was performed. As shown in Fig. 2B, NDRG2 was expressed mainly in the cytoplasm and could hardly be detected in nucleus before OGD exposure. After exposure to OGD, the NDRG2 expression in both nucleus and cytoplasm was sharply increased.

### NDRG2 Down-regulation Alleviated the OGD-induced Astrocytes Apoptosis

**Figure 3:**
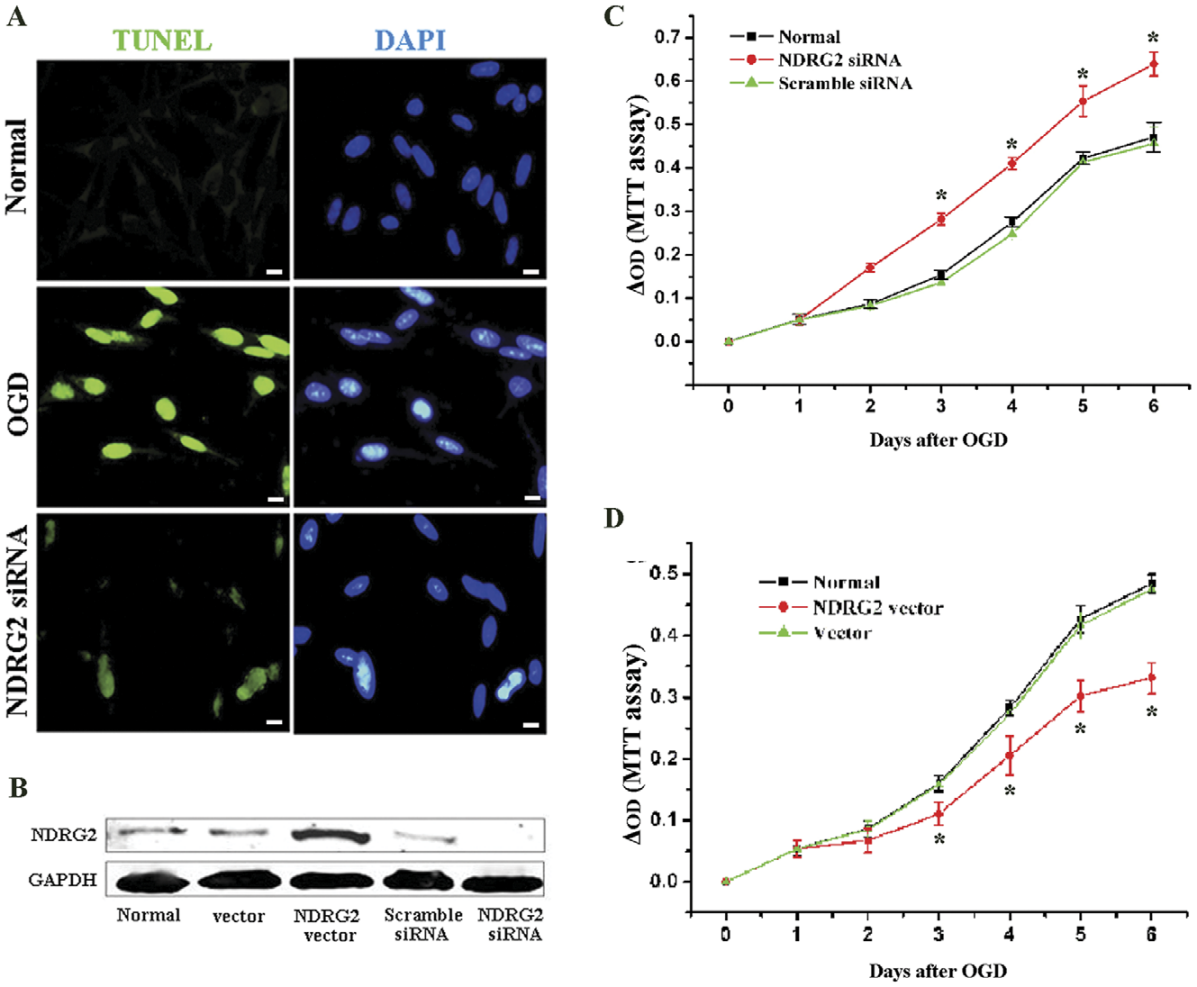
Effect of NDRG2 expression on cellular proliferation and apoptosis after OGD exposure. (**A**) TUNEL (green) and DAPI (blue) double-staining was used to test the apoptosis of C6-originated astrocytes at the time of 24 h after OGD. NDRG2 down-regulation with NDRG2-specfic siRNA greatly reduced the enhancement of TUNEL and DAPI signals after OGD. Scale bar = 10 µm. (**B**) The over-expression and silencing systems of NDRG2 were constructed and verified by Western-blotting. From left to right, the C6-originated astrocytes were kept normal (Normal), or transfected with the empty pEGFP-C1 vector (vector), the pEGFP-C1 vector expressing NDRG2 (NDRG2 vector), scramble siRNA (Scramble siRNA), and NDRG2-specific siRNA (NDRG2 siRNA) in order. (**C**) At the day 3, 4, 5, 6, 7 after OGD exposure, the astrocytes transfected with NDRG2-specific siRNA presented improved proliferation, compared with normal cells and those transfected with scramble siRNA. (**D**) At the day 3, 4, 5, 6, 7 after OGD exposure, the astrocytes with over-expressed NDRG2 presented restrained proliferation, compared with normal cells and those transfected with empty pEGFP-C1 vector. All data were presented as the mean ± SD of three independent experiments. ANOVA, *P<0.05 *vs*. normal.

In the previous study, we observed the NDRG2 signals co-localized with some TUNEL-positive cells after transient focal cerebral ischemia [7]. In this study, we detected the occurrence of apoptosis after OGD exposure in the astrocytes originated from C6 glioma cells. As shown in Fig. 3A, the normal astrocytes obtained stronger TUNEL-positive staining after OGD exposure indicating a process of OGD-induced apoptosis in astrocytes. However, the down-regulation of NDRG2 with small interfering RNA (NDRG2 siRNA) alleviated the OGD-induced enhancement in TUNEL-positive staining.

The over-expression and silencing systems of NDRG2 were verified by Western-blotting (Fig. 3B). To investigate the functional role of NDRG2 under OGD conditions, cells were respectively transfected with pEGFP-C1 constructs expressing NDRG2 (NDRG2 vector), pEGFP-C1 (vector), NDRG2-specific siRNA (NDRG2 siRNA) or scramble siRNA before exposed to OGD. Compared with normal cells or those transfected with scramble siRNA, the cells with down-regulated NDRG2 revealed a stronger increment of MTT optical density (OD), i.e., a pro-proliferative effect since day 3 up to day 6 after exposed to OGD (Fig. 3C). On the contrary, the cells with up-regulated NDRG2 expression exhibited an anti-proliferative effect (Fig. 3D).

### NDRG2 Promoted Up-regulation of Bax in Astrocytes after OGD Exposure

**Figure 4:**
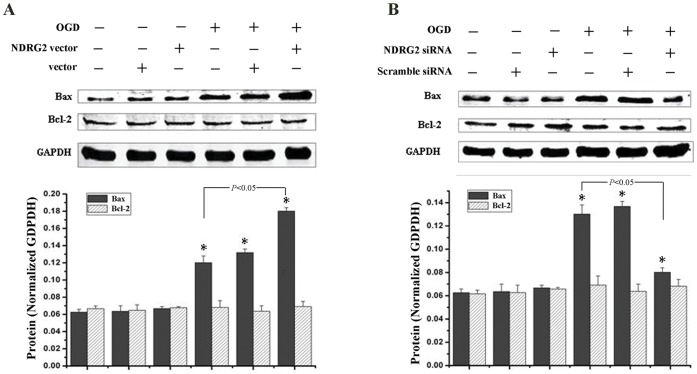
Effect of NDRG2 expression on Bax and Bcl-2 after OGD. The C6-originated astrocytes were transfected with pEGFP-C1 constructs expressing NDRG2 (NDRG2 vector), empty pEGFP-C1 (vector), NDRG2-specific siRNA (NDRG2 siRNA) or scramble siRNA before OGD. The levels of Bax and Bcl-2 were measured by Western-blotting at the time of 24 h after OGD exposure. (**A**) OGD induced a higher Bax expression, which could be further improved by NDRG2 over-expression. Neither NDRG2 over-expression nor OGD stimuli had effect on the Bcl-2 expression. (**B**) NDRG2 silencing with NDRG2-specific siRNA greatly suppressed the OGD-induced Bax uprising, and had no impact on the Bcl-2 expression. All data were presented as the mean ± SD of three independent experiments. ANOVA, *P<0.05 vs. normal.

Bax and Bcl-2, two apoptosis-related proteins, were measured in this study to verify the role of NDRG2 in OGD-induced apoptosis. As shown in Fig. 4, Bax expression was significantly increased after OGD exposure, while Bcl-2 expression was kept unchanged compared to in normal astrocytes. The higher Bax/Bcl-2 ratio induced by OGD was aggravated by NDRG2 over-expression (NDRG2 vector) (Fig. 4A), but attenuated by NDRG2 silencing (NDRG2 siRNA) (Fig. 4B). The change in Bax/Bcl-2 ratio in relation to NDGR2 expression further supported the role of NDRG2 in OGD-induced apoptosis.

### Down-regulation of p53 Reduced OGD-induced Astrocytes Apoptosis

**Figure 5:**
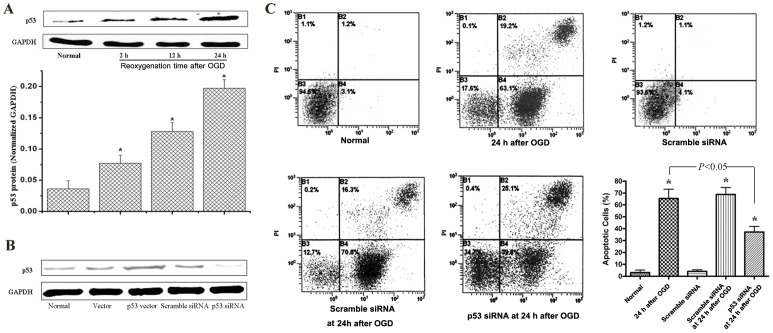
p53 down-regulation suppressed the OGD-induced cellular apoptosis. (**A**) In C6-originated astrocytes, p53 appeared a time-dependent uprising after OGD exposure, which started at the time of 2 h and then peaked at the time of 24 h after OGD. Student’s *t* test. (**B**) The over-expression and silencing systems of p53 were constructed and verified by Western-blotting. From left to right, the C6-originated astrocytes were kept normal (Normal), or transfected with the empty pEGFP-C1 vector (vector), the pEGFP-C1 vector expressing p53 (p53 vector), scramble siRNA (Scramble siRNA), and p53-specific siRNA (p53 siRNA) in order. (**C**) The effect of p53 on the OGD-induced apoptosis in astrocytes was evaluated by flow cytometry analysis. As presented in histogram, p53 silencing with p53 siRNA greatly reduced the percentage of apoptotic cells at the time of 24 h after OGD. All data were presented as the mean ± SD of three independent experiments. ANOVA, *P<0.0*5 vs*. normal.

p53 is usually regarded as a pro-apoptotic factor. In this study, we tested the role of p53 in OGD-induced astrocytes apoptosis. We found that the up-regulation of p53 protein also appeared in a time-dependent manner after OGD exposure, similar to the OGD-induced change in NDRG2 (Fig. 5A). The p53 protein level began to increase at the time of 2 h after OGD exposure and reached a peak at 24 h.

The over-expression and silencing systems of p53 were verified by Western-blotting (Fig. 5B). The astrocytes were respectively transfected with pEGFP-C1 constructs expressing p53 (p53 vector), pEGFP-C1 (vector), p53-specific siRNA (p53 siRNA) or scramble siRNA before exposed to OGD. The flow cytometry analysis showed that p53 silencing greatly reduced the percentage of apoptotic cells and displayed an anti-apoptotic effect (Fig. 5C).

### NDRG2 was Up-regulated in a p53-associated Manner after OGD Exposure

**Figure 6:**
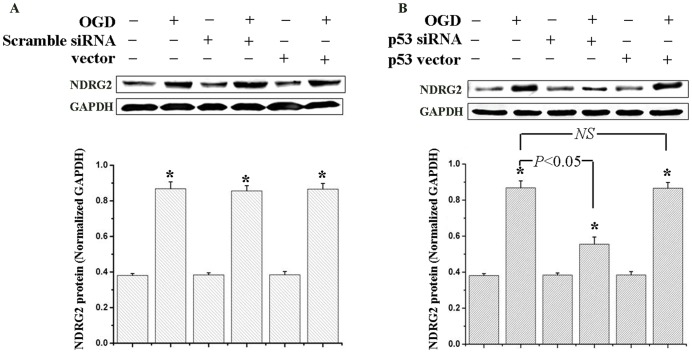
The role of p53 in OGD-induced NDRG2 up-regulation. In differently transfected astrocytes, the NDRG2 expression was detected at the time of 24 h after OGD exposure by Western-blotting. (**A**) Compared to normal cells after OGD, the astrocytes transfected with either scramble siRNA of p53 or empty vector presented a similar uprising of NDRG2. Without an OGD stimulus, the NDRG2 up-regulation would not happen. (**B**) The p53 silencing obviously suppressed the up-regulation of NDRG2 after OGD, and its over-expression did not further improve the NDRG2 increase. All data were presented as the mean ± SD of three independent experiments. ANOVA, *P<0.05 *vs*. normal.

With the help of the over-expression and silencing systems of p53, we investigated the role of p53 in OGD-induced NDRG2 up-regulation. As shown in Fig. 6A, those astrocytes transfected with either scramble siRNA of p53 or empty vector, demonstrated a similar NDRG2 uprising compared to what happened in normal astrocytes treated with OGD. The p53 silencing obviously suppressed the up-regulation of NDRG2, although the level was still higher than that in normal cells. However, p53 over-expression could not further increase the NDRG2 level after OGD (Fig. 6B). These findings pointed out that OGD-induced NDRG2 expression was associated with p53.

## Discussion

In past majority of studies in cerebral ischemic injury, the attention had been mainly focused on the fate of neurons. The role of astrocytes had been neglected for a long time, despite of the fact that the number of astrocytes in CNS is over fivefold of neurons. In the recent 25 years, a revolutionary understanding has been developed with more and more focus in the physiology and pathology of astrocytes. Nowadays astrocytes are considered as “the principal housekeeping cells” in CNS [13]. Structurally connecting the entire CNS, astrocytes also perform supportive functions such as blood-brain barrier formation, synaptic transmission [14], regulation of blood flow [15], maintenance of the homeostasis in synaptic interstitial fluid [16], [17], and energy metabolism [18]. Therefore, the intact functional status of astrocytes is crucial for neuronal survival after various injuries.

In the previous works, it has been confirmed by separate teams that NDRG2 is localized in astrocytes in healthy cerebrum [19], [20]. Our previous study also reported that the NDRG2 signals were further enhanced in reactive astrocytes of penumbra after transient middle cerebral artery occlusion (MCAO) in rats [7]. In the present study, we employed IL-6-differentiated C6 glioma cells as mature astrocytes and injured them in an OGD model, as referred previously [21], [22]. After OGD 4h followed by re-oxygenation, we observed a significant increase of both TUNEL-positive staining and NDRG2 expression in a time-dependent manner, parallel to the finding in previous *in vivo* study [7], indicating that this OGD model in IL-6-differentiated C6 glioma cells could mimic the I/R injury induced by transient focal cerebral ischemia in rats.

The phenomenon of NDRG2 signals co-localized with TUNEL-positive cells in ischemic penumbra suggested that NDRG2 might be involved in cellular apoptosis induced by ischemia [7]. In the present study, we constructed NDRG2 over-expression and silencing systems to verify the role of NDRG2 in cellular apoptosis and proliferation. The results showed that NDRG2 over-expression could inhibit the proliferation of astrocytes after OGD, while its silencing made an opposite effect. Moreover, over-expression of NDRG2 enhanced the increase of Bax/Bcl-2 ratio after OGD while NDRG2 silencing attenuated such an increase. Bax and Bcl-2 are two important members of Bcl-2 family that is closely associated with cellular fate [23]. Bcl-2 acts as an anti-apoptotic signal and Bax is pro-apoptotic. Therefore, the Bax/Bcl-2 ratio is considered as the switch to determine the cell death [24]. Our present data showed that the decreased survival rate in up-regulated NDRG2 astrocytes after OGD exposure was due to the pro-apoptotic effect of Bax. On the other hand, it was interesting that the expression of Bcl-2 was uninfluenced, no matter when NDRG2 was in a level of up- or down-regulation. The interaction between NDRG2 and Bcl-2 still needs to be clarified.

So far we have demonstrated that NDRG2 promoted the cellular apoptosis after OGD. Our findings are in line with that reported by Wang *et al* in A549 cells [25], however, inconsistent with that reported by Liu *et al* in cervical cancer Hela cells [26]. Liu and colleagues found that the over-expression of NDRG2 resulted in increased surviving rate, decreased percentage of apoptotic cells and lowered Bax/Bcl-2 ratio after irradiation exposure. On the contrary, NDRG2 silencing contributed to decreased cells survival, increased apoptosis and higher Bax/Bcl-2 ratio. These might be the consequences of different inherent characteristics among different types of tumor cell and different mechanisms associated with various injury-causing factors, such as irradiation, OGD, and ischemia, etc. It still needs to be further investigated.

In resting status, NDRG2 expression was observed mainly in the plasma membrane and cytoplasm [19], [20], [27]. Upon cell stress like hypoxia and ischemia, the translocation of NDRG2 from the cytoplasm to the nucleus will occur [7], [25]. Using the technique of cell fraction assay, we confirmed this phenomenon quantitatively. Such a stress-accompanying change generally indicates the activation of intracellular signaling pathways. The mechanisms and effects of NDRG2 nuclear translocation are still unknown. Present data has not shown that there exists any nuclear localization signal in NDRG2 protein, which is the most common form of nuclear import elements. Therefore, it is presumed that NDRG2 might have its own motif to guide its nuclear translocation under particular conditions. Wang *et al* found the segment of residue 101–178 in NDRG2 pivotal to its translocation [25]. Recently, Hwang *et al* demonstrated that helix α6 of hNDRG2 might contribute to the translocation, based on the knowledge of the three-dimensional crystal structure [28].

p53 is the master regulator of cell death by inducing apoptosis and its biological effects are mainly explained by its activity as a transcription factor [8]. Bax is one of its downstream target genes associated with pro-apoptotic effect. It was documented that p53 could regulate the Bax transcription in focal ischemia and experimental Parkinson’s disease [29], [30]. To verify whether OGD-induced NDRG2 up-regulation is associated with p53, we constructed p53 over-expression and silencing systems. It appeared that p53 silencing obviously suppressed the up-regulation of NDRG2 after OGD. Interestingly, over-expression of p53 did not further strengthen the uprising of NDRG2 after OGD. From these data, we can demonstrate that OGD-induced NDRG2 uprising was regulated by p53 expression, which was consistent with Liu’s report [31]. It is noteworthy that over-expression of p53 could not lead to a higher NDRG2 expression than that observed in simply OGD-treated cells. Several upstream regulators acting on NDRG2 promoter were reported, such as HIF-1α and p53 transactional activated NDRG2 while Myc transactional suppressed NDRG2 [25], [31], [32]. On the other hand, although p53 is commonly labeled as a pro-apoptotic gene, it could transcriptionally activate some anti-apoptotic genes, such as HB-EGF, DcR1 and DcR2 [33], [34], [35]. Taken all these into consideration, the p53 pathway is a complex network.

In conclusion, NDRG2 is a novel regulator of apoptosis. It plays an important role in the p53-related pro-apoptotic effect when the astrocytes originated from C6 glioma cells are exposed to OGD. And the pro-apoptotic effect of NDRG2 is independent of Bcl-2. Our findings bring insight to the roles of NDRG2 in ischemic-hypoxic injury and provide potential targets for future clinical therapies on stroke.

## Materials and Methods

### Cell Culture

Rat glioma cell line, C6 was obtained from the American Type Culture Collection (ATCC, Manassas, Virginia, USA) and maintained at 37°C in a humidified atmosphere containing 5% CO_2_. Cells were cultured in RPMI 1640 medium, supplemented with 10% fetal bovine serum. Experiments were carried out 24 h after cells were seeded. Recombinant Rat IL-6 was purchased from PeproTech (New Jersey, USA). C6 cells were plated at a density of 1×10^6^ cells/well in a six-well dish and treated with 100 ng/ml IL-6 to induce cell differentiation for 24 h [36].

### Oxygen-glucose Deprivation (OGD)

Oxygen-glucose deprivation model was made as described previously [37]. The original medium was removed and the cells were washed with oxygen and glucose-free Earle’s balanced salt solution (EBSS, pH 7.4). The cultures were then placed in fresh oxygen and glucose-free EBSS and held in an incubator containing 95% (v/v) N_2_ and 5% (v/v) CO_2_ at 37°C for 4 hours. Then glucose was added in, and the cells were returned to normal condition for additional 2 h, 12 h or 24 h (i.e. reoxygenation 2 h, 12 h and 24 h, respectively).

### Plasmid Construction

DNA fragments encoding NDRG2 and p53 were PCR-amplified using a human brain cDNA library as template with the following primers: 5′-GAATTCTATGGCAGAGCTTCAGGAGGT-3′ and 5′-GGATCCTCAACAGGAGACTTCCATGGT-3′ for NDRG2, and 5′-GAATTCTATGGAGGATTCACAGTCGGATA-3′ and 5′-GGATCCTCAGTCTGAGTCAGGCCCCA-3′ for p53, respectively. The DNA fragments were cloned into the EcoR/BamH sites of the pEGFP-C1 vector (Clontech, Palo Alto, CA). Expression vectors were transfected into cells with Lipofectamine 2000 (Invitrogen) according to the manufacturer’s instructions.

### Cells Treated with siRNA for NDRG2 or p53

All siRNA oligonucleotides were purchased from QIAGEN Company (Duesseldorf, Germany). The sequences targeted to RAT NDRG2 (NM_133583) are 5′-GCAUCCUGCAGUACUUAAATT’ and 5′-UUUAAGUACUGCAGGAUGCAA-3′. The sequences targeted to RAT p53 (NM_030989) are 5′-CAGCGACAGGGUCACCUAATT’ and 5′-UUAGGUGACCCUGUCGUCGCUGTG-3′. The siRNAs were transfected into cells with Lipofectamine 2000 (Invitrogen).

### Western Blotting

For Western blotting analysis, cells were harvested from 60-mm dishes and were lysed in modified radioimmunology precipitation assay buffer. Protein concentration was measured by bicinchoninic acid (BCA) protein assay (Pierce, Rockford, IL, USA). Proteins were separated by sodium dodecyl sulfate polyacrylamide gel electrophoresis (SDS-PAGE) and transferred to Hybond ECL nitrocellulose membranes (Amersham Bio-sciences, Little Chalfont, Buckinghamshire, UK). Anti-NDRG2 mouse monoclonal antibody (1∶1000; Abnova Corporation, Epitomics, USA), and Anti-Bcl2 mouse monoclonal antibody (1∶500; Santa Cruz, USA), anti-GAPDH rat monoclonal antibody (1∶500; Boster, Wuhan, China) were used for immunoblotting. To visualize primary antibody-bound proteins, secondary antibodies conjugated to IRDye800 (1∶20000; Rockland Inc., Gilbertsville, PA, USA) and an Odyssey infrared imaging system (LI-COR Inc., St Lincoln, NE, USA) were used.

### RT-PCR

For reverse transcriptase RT-PCR analysis, cells were harvested from 60-mm dishes and total RNA was immediately isolated from each sample using TRIZOL reagent (Invitrogen, Carlsbad, CA, USA) and then quantified. Two micrograms of total RNA was reverse-transcribed using reverse transcriptase (Promega, Madison, WI, USA) according to the manufacturer’s instructions. All PCR experiments were performed using Taq polymerase (Promega) with the following primers: 5′-TTGCTACCCTAACCTTGACC-3′ and 5′-TCCCGTTCGACTTTCTTTT-3′ for rat NDRG2, and 5′-GCAAATTCAACGGCACAGTCAAGG-3′ and 5′-ATCACGCCACAGCTTTCCAGAGG-3′ for rat glyceraldehyde-3-phosphate dehydrogenase (GAPDH) control. The PCR products were resolved on 1% agarose gel containing ethidium bromide and bands were visualized in ultraviolet light.

### Immunofluorescent Double-labeling Staining

Fisherbrand coverglasses were prepared and nonspecific antibody-binding sites were blocked with 1% bovine serum albumin in phosphate-buffered saline (1% BSA-PBS). The coverglasses were incubated with anti-NDRG2 mouse monoclonal antibody (1∶200) and anti-GFAP rabbit monoclonal antibody (1∶300; DakoCytomation, Glostrup, Denmark) in 1% BSA-PBS overnight at 4°C. The coverglasses were then washed with TBS and incubated with anti-mouse FITC tagged secondary antibody (1∶200; CWBIO, Peking, China) and anti-rabbit CY3 tagged secondary antibody (1∶200; CWBIO, Peking, China) for 2 h at room temperature. At last DAPI (1 ng/µL) was used to stain nucleus. The coverglasses were mounted with 50% glycerol for examination under a fluorescence microscope.

### TUNEL

Apoptosis was quantified using a commercially available fluorescent terminal deoxynucleotidyl transferase nick-end labeling (TUNEL) kit, in accordance with the manufacturer’s protocol (Roche Diagnostics Corporation, Indianapolis, IN, USA). And then the Fisherbrand coverglasses were stained with DAPI (1 ng/µL). The sections were mounted with 50% glycerol for examination under a fluorescence microscope.

### Cell Fraction Assay

At the time of 24 h after cells were exposed to OGD, nuclear extracts were prepared as described in the protocol of NE-PER nuclear and Cytoplasmic Extraction Reagents (Pierce, Rockford, IL). Briefly, 1×10^7^ cells were washed twice with ice-cold PBS and added to 200 µl of ice-cold cytoplasmic extraction reagent I, incubated on ice for 10 min, added to 11 µl of ice-cold cytoplasmic extraction reagent II, and incubated on ice for 1 min; the tube was centrifuged for 5 min at maximum speed in a microcentrifuge (16,000 *g*). The supernatant fraction (cytoplasmic extract) was immediately transferred to a clean pre-chilled tube and 100 µl of ice-cold nuclear extraction reagent was added into the insoluble fraction by vortexing for 15 s every 10 min for a total of 40 min. The tube was centrifuged at maximum speed in a microcentrifuge for 10 min. The nuclear extract fraction was moved to a clean pre-chilled tube. All extracts were analyzed by Western blotting.

### MTT Assay

The cells were seeded into 96-well plates at a starting density of 1×10^3^ cells/well in triplicate. At each time point, the cells were washed and incubated with tetrazolium salt (MTT, 100 mg/ml, Sigma) at 37°C for 4 hours. The supernatant was removed, and DMSO was added for 150 µl per well. The absorbance (OD) of the reaction solution at 570 nm was recorded. Each experiment was performed for three times, and the values were reported as the mean ± SD.

### Flow Cytometry Analysis

The percentage of apoptotic cells was detected by flow cytometry analysis. Cells were harvested and washed with PBS. Cell death was measured using two-color analysis of fluoresce in isothiocyanate-labeled annexin V (Roche Applied Science) binding and propidium iodide (PI) uptake with Becton Dickinson fluorescence-activated cell sorter (FACS) apparatus.

### Statistical Analysis

Statistical analysis was performed with SPSS software (version 10.0; SPSS, Chicago, IL). The differences among groups were analyzed by ANOVA and the difference between two groups was analyzed by two-tailed Student’s *t* test. Results are presented as mean ± SD from at least three independent experiments unless otherwise stated. Statistical significance was defined as *P*<0.05 and histograms were prepared with Origin 6.0 (Microcal Software, Inc., Northampton, MA).
